# White-toothed shrews (Mammalia, Soricomorpha, *Crocidura*) of coastal islands of Vietnam

**DOI:** 10.3897/zookeys.207.3237

**Published:** 2012-07-11

**Authors:** Alexei V. Abramov, Anna A. Bannikova, Viatcheslav V. Rozhnov

**Affiliations:** 1 Zoological Institute, Russian Academy of Sciences, Universitetskaya nab. 1, Saint Petersburg 199034, Russia; 2 Lomonosov Moscow State University, Vorobievy Gory, Moscow 119992, Russia; 3 A.N. Severtsov Institute of Ecology and Evolution, Russian Academy of Sciences, Leninskii pr. 33, Moscow 119071, Russia; 4 Joint Vietnam-Russian Tropical Research and Technological Centre, Nguyen Van Huyen, Nghia Do, Cau Giay, Hanoi, Vietnam

**Keywords:** *Crocidura fuliginosa*, *Crocidura attenuata*, *Crocidura phuquocensis*, biogeography, Cat Ba, Con Dao, Phu Quoc, SE Asia

## Abstract

New findings of the white-toothed shrews (*Crocidura* spp.) from offshore islands of Vietnam are reported. The species identifications have been confirmed by the analysis of complete mitochondrial cytochrome *b* gene (1140 bp). *Crocidura phuquocensis* is the only species found in the Phu Quoc Island. *Crocidura fuliginosa* has been recorded from two islands of the Con Dao Archipelago (Con Son and Bai Canh). The occurrence of *Crocidura fuliginosa* in Vietnam has been genetically confirmed for the first time. *Crocidura attenuata* has been collected from the Cat Ba Island for the first time, and this finding corresponds well with the proposal that the species’ distribution is confined to the north and east of the Red River only.

## Introduction

Biodiversity of Southeast Asian islands has often been the focus of intensive studies by systematists, evolutionary biologists and biogeographers (Meijaard 2003; Esselstyn and Brown 2009; Esselstyn et al. 2009; Esselstyn and Oliveros 2010). Such studies of insular faunas of terrestrial mammals make it possible to examine geographic and temporal processes of diversification. The species distribution and richness of island faunas are determined by colonization and extinction events and are largely dependent on geographical characteristics of the islands, particularly their size and isolation.

Despite its small size, Vietnam has a very long coastline (near 3500 km) and is surrounded by more than 3000 islands. The majority of Vietnamese coastal islands are situated within a shallow shelf. Two main stages of the geological history of this area can be recognized (Korotky et al. 1995). At the first stage, most parts of the modern South China Sea shelf were continental. During following transgressions the continent was submerged and many coastal islands developed. These islands were from time to time connected to the continent during repeated sea level fluctuations in the Pleistocene. Island communities off mainland Vietnam may reflect dispersal and vicariance events initiated by climate change.

**Figure 1.**
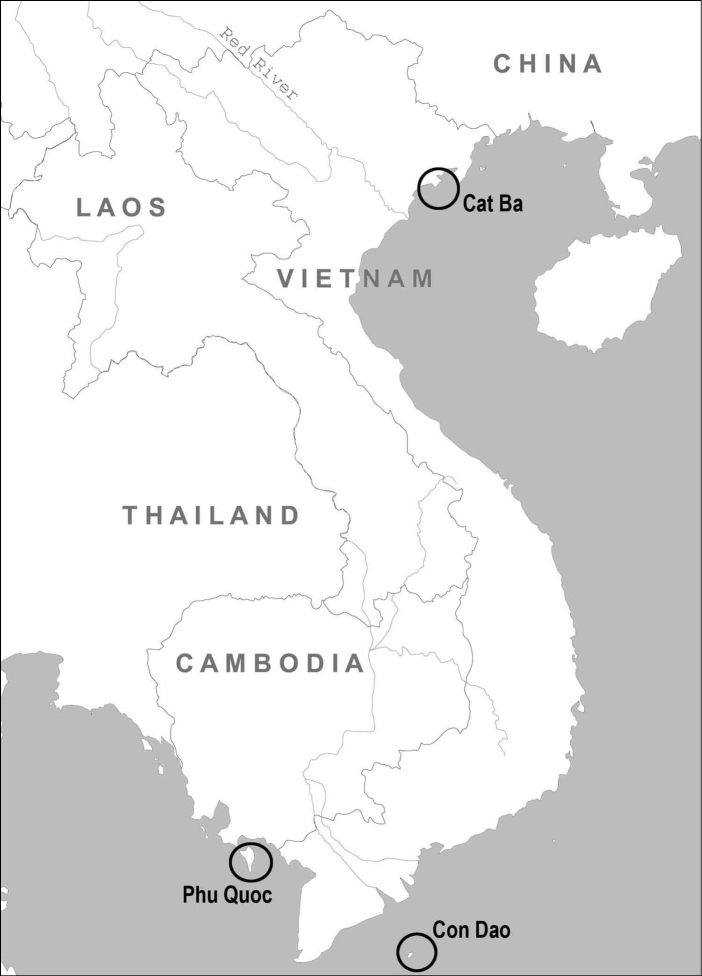
Map of Vietnam. Location of islands studied is shown.

Most mammal surveys of Vietnamese islands have been devoted to the study of rodents and large mammals (Van Peenen et al. 1970, Kuznetsov and Anh 1992, Kuznetsov 2000). Shrews remain poorly studied in these areas. A few biodiversity surveys were conducted by the Joint Vietnam-Russian Tropical Research and Technological Centre (VRTC) on coastal islands of Vietnam (Fig. 1) during 2003-2011. In this paper, we have summarized the results of the study of white-toothed shrews resulting from these surveys.

## Studied area

Phu Quoc is the largest Vietnamese island (it covers ca. 562 km^2^) lying in the Gulf of Thailand, ca. 15 km south of the coast of mainland Cambodia. Primary lowland tropical forests still cover the northeastern part of the island. The first mammalogical survey of Phu Quoc was carried out of the VRTC (Abramov et al. 2007a) in the period of 25 November - 20 December, 2003. In total, 105 trap-nights were conducted using pitfall traps (plastic buckets 40×30 cm) located in a few different biotopes.

The Con Dao Archipelago is situated in the monsoon belt of the South China Sea at about 90 km off mainland Vietnam. Con Son, formerly known as Pulo Condor, consists of the largest (ca. 52 km^2^) island of archipelago, surrounded by 14 smaller islets. The topography of Con Son Island is mountainous, and is dominated by the granite ridge running from south-west to north-east and is covered by primary tropical forest. A biodiversity survey of Con Son was conducted by the VRTC from 26 May to 12 June, 2010. Small mammal trapping was conducted using plastic buckets (25×20 cm) and glasses (13×9 cm) as pitfall traps. Trapping took place for a total of 1237 trap-nights, distributed unequally between 13 survey sites.

Cat Ba is the largest of hundreds of islands that comprise the Cat Ba Archipelago and is located at the southeastern edge of Ha Long Bay in northern Vietnam. Cat Ba Island lies approximately 30 km east of Hai Phong city in northern Vietnam and has a surface area of 285 km^2^. The landscape of Cat Ba is dominated by limestone karst with alternating narrow valleys running along the northeast-southwest line. The main natural vegetation type on Cat Ba consists of moist tropical forest on limestone karst, however, in large areas it is now replaced by limestone scrub or bare rocks. Fieldworks were carried out by the VRTC in the central part of Cat Ba Island from 10 to 25 October, 2011. In total, 650 trap-nights were conducted using pitfall traps (plastic glasses of 13×9 cm) located in five different biotopes.

## Material and methods

A total of thirteen *Crocidura* specimens were collected during the three aforementioned island surveys. Specimens were fixed in 70% ethanol. Tissue samples were preserved in 96% ethanol. Skulls were extracted and cleaned from many specimens. Standard external body measurements (head and body length, tail length, hind foot length) were taken in the field. Weight was measured in grams. Voucher specimens are kept in the Zoological Institute of the Russian Academy of Sciences (Saint-Petersburg, Russia).

Genomic DNA was isolated from ethanol-fixed kidney or muscles by proteinase K digestion, phenol-chloroform deproteinization and isopropanol precipitation (Sambrook et al. 1989).

The complete mitochondrial cytochrome *b* gene (*cytb*, 1140 bp) was amplified by PCR with the primer combination and conditions for *cytb* amplification as in Bannikova et al. (2011). Primers L14728_Cr (5`-GACATGAAAAATCATCGTTGTTCTTCAAC-3`) and H1310_Cr (5`-GAATATCAGCTTTGGGTGYTGATGGTGG-3`) were used for amplification of the whole *cytb* gene (1140 bp), and primers Cro_481b (5`-ACGGAAAAGCCTCCTCAGATTCATTCTAC-3`) and L363A (5`-CGCAGTTATAGCCACCGCCTTTATAGG-3`) were used for sequencing and amplification of short parts of the gene if necessary. Sequencing with each primer was performed by the ABI 3100-Avant autosequencing system using ABI PRISM®BigDyeTM Terminator v. 3.1. *Cytb* gene sequences were aligned by eye using BioEdit v.7.0.5.3 (Hall 1999). The final alignment of the mitochondrial gene included 1140 bp. Diversity patterns of *cytb* sequences were assessed using maximum parsimony (MP) and neighbour joining (NJ) methods with the help of PAUP* version 4.0b10 (Swofford 1998) based on pairwise *p*-distance matrix. To assess clade support, 1000 bootstrap pseudoreplicates were analyzed.

GenBank accession numbers for the original sequences used in the study are JX181934-JX181941.

We also included *cytb* sequence data from several earlier studies (Ruedi et al. 1998; Bannikova et al. 2006, 2011; Dubey et al. 2008; Esselstyn et al. 2009; Lavrenchenko et al. 2009) to place the shrews from Vietnam into a regional phylogeographic and phylogenetic context.

## Results

### Crocidura fuliginosa

(Blyth, 1855)

http://species-id.net/wiki/Crocidura_fuliginosa

We have found this species on Con Son Island only. A single specimen was collected near Nui Nha Ban on the north slope of Nui Chua Mt. in the central part of island. The pitfall traps (08°42'49"N, 106°37'13"E) were set in moist primary forest at an elevation of 250 m asl. Despite considerable trapping efforts (more than 1200 trap-nights) we collected only one specimen. This species was firstly reported from Con Son by Van Peenen et al. (1970). A single adult male (USNM 357348, Smithsonian Institution) was caught in a small Sherman trap at the side of a trail leading to Nui Nha Ban, at the elevation of 80 m asl. Another specimen (ZMMU S-144368, Zoological Museum of Moscow University) was collected in 1987 in the forest of Bai Canh Islet located just 1 km eastward of the Con Son Island.

This is a large, long-tailed *Crocidura*; tail 79% of head and body length, on average. Means and extremes of measurements (in mm) from 3 adults are: head and body length, 87.0 (83–90); tail length, 68.3 (67–71); hind foot length, 15.7 (15–17); weight (n=1), 16.7 g.

**Figure 2.**
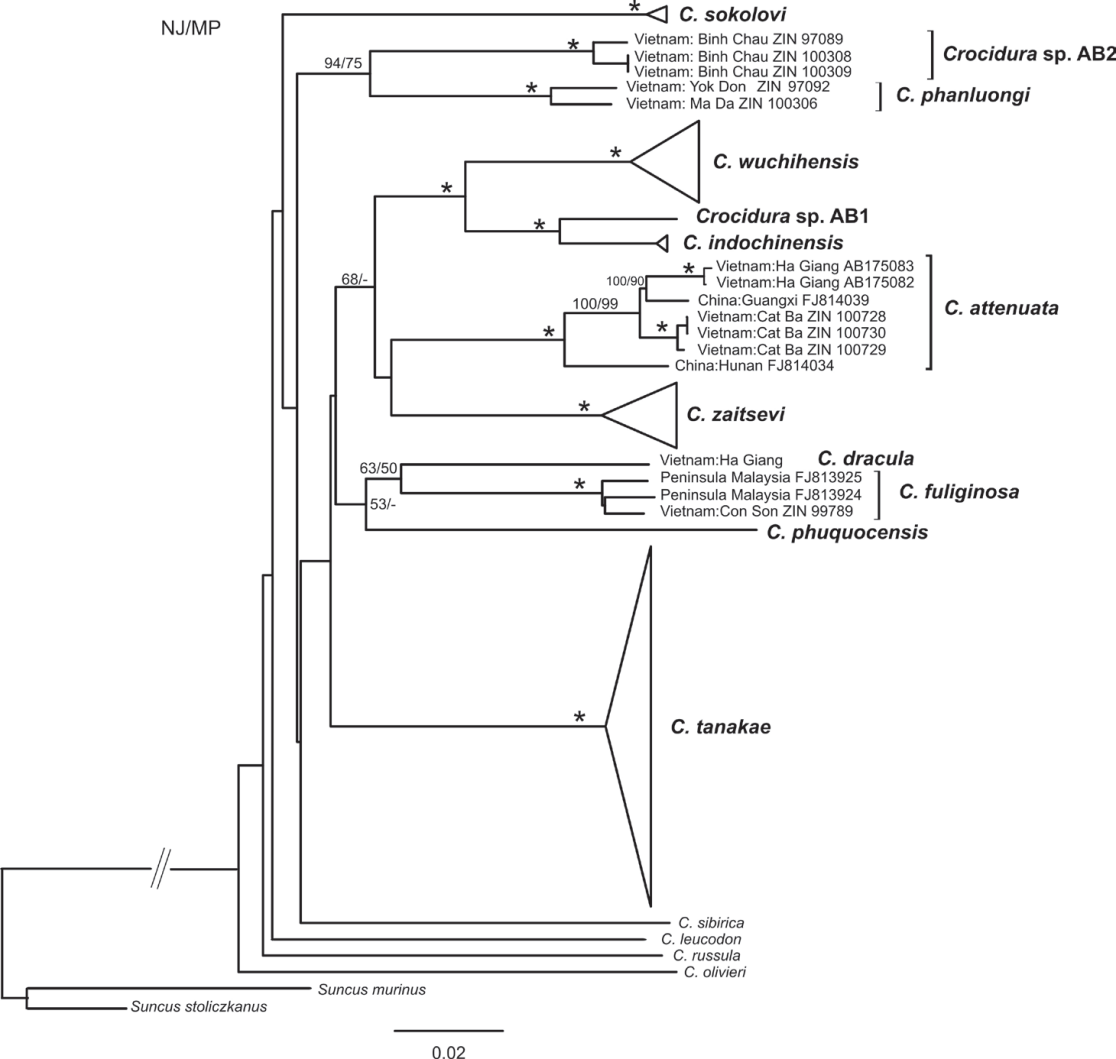
The NJ tree for the *cytb* gene. The bootstrap values (³50%) obtained from 1000 replications in NJ/MP analyses are presented above the branches. «*» denotes bootstrap support of 100% in both NJ and MP analyses; «-» indicates support values of less than 50%. *Suncus murinus* and *Suncus stoliczkanus* are used as outgroup.

The mtDNA analysis suggests that specimen from the Con Son Island belongs to *Crocidura fuliginosa* (Fig. 2).

Many papers listed *Crocidura fuliginosa* as being widespread in mainland Vietnam (Heaney and Timm 1983; Huynh et al. 1994; Kuznetsov 2006; Can et al. 2008; Jenkins et al. 2009). A recent comparative study of mtDNA (Bannikova et al. 2011) did not confirm its occurrence in Vietnam. Specimens from northern Vietnam (Ha Giang Province) are very different from the shrews taken from the Cameron Highlands (Peninsular Malaysia) assigned to this species. Moreover, the northern Vietnamese specimens are close to the shrews from Yunnan, southern China. A major distinction between Yunnan specimens and *Crocidura fuliginosa* from Peninsular Malaysia was also found by Dubey et al. (2008) in the analysis of nuclear genes. Bannikova et al. (2011) proposed to re-establish the name *Crocidura dracula* for the large white-toothed shrews from northern Vietnam and southern China. This taxon was described by Thomas (1912) from southern Yunnan. According to Allen (1938) and Ellerman and Morrison-Scott (1951), this species is distributed across southern China and adjacent Indochina. Jenkins (1976) considered *dracula* a subspecies of *Crocidura fuliginosa*, and was followed in this opinion by various authors (Heaney and Timm 1983; Jiang and Hoffmann 2001; Hutterer 2005). Based on the data from mitochondrial DNA, the name *fuliginosa* was provisionally restricted to the shrews from the southern part of Southeast Asia, including Malaysia and southern Myanmar (Bannikova et al. 2011). A comparison of *cytb* sequences suggests that specimen from the Con Son Island belongs to this haplogroup (Fig. 2). This is a first genetically confirmed record of *Crocidura fuliginosa* from Vietnam.

The occurrence of *Crocidura fuliginosa* sensu stricto in mainland Vietnam is still questionable (see also Heaney and Timm 1983). Jenkins et al. (2009) have mentioned museum specimens of *Crocidura fuliginosa* (= *dracula* sensu Bannikova et al. 2011) from Lao Cai in northern Vietnam and Yunnan in southern China only. Two other records mentioned by Jenkins et al. (2009) were based on survey reports, not on museum voucher specimens. One of them (Trai et al. 1999), reporting *Crocidura fuliginosa* from Ngoc Linh Mt. in Kon Tum Province, central Vietnam, was based on a visual observation only. Another location mentioned (see Jenkins et al. 2009) is Nui Bi Doup, Lam Dong Province, southern Vietnam. In 2004 and 2006, during mammal surveys in Ngoc Linh Mt. conducted by the VRTC, we collected 116 shrews of three *Crocidura* species (Abramov et al. 2007b; Rozhnov and Abramov 2009). Two of these species, *Crocidura sokolovi* and *Crocidura zaitsevi*, were new to science (Jenkins et al. 2007); the third one was *Crocidura tanakae* (see Bannikova et al. 2011). Several biodiversity surveys conducted by the VRTC in Nui Bi Doup area in 2002-2009 (Abramov et al. 2010) yielded more than 100 shrews of three *Crocidura* species, including *Crocidura tanakae*, *Crocidura indochinensis* and *Crocidura zaitsevi* (see Bannikova et al. 2011). However, we documented no specimens of *Crocidura fuliginosa*, neither in the Ngoc Linh nor in the Bi Doup areas.

### Crocidura phuquocensis

Abramov, Jenkins, Rozhnov et Kalinin, 2008

http://species-id.net/wiki/Crocidura_phuquocensis

Five adult white-toothed shrews were collected in the northern part of Phu Quoc Island (10°22'53"N, 104°00'19"E), 5 km west of Bai Thom Village, near the road Duong Dong-Bai Thom, close to the northern slope of Mount Chua area, at the elevation of about 30 m asl. Pitfall traps were set up in the dipterocarp forest, near tumbled down trees, at the bottom of a mountain. On the basis on these specimens, *Crocidura phuquocensis* was described by Abramov et al. (2008).

This is a medium-sized *Crocidura*, with a moderately long tail; tail 75% of head and body length, on average. Means and extremes of measurements (in mm) from 5 adults are: head and body length, 76.2 (68–72); tail length, 52.4 (49–59); hind foot length, 12.1 (12–12.5).

The validity of this species was recently confirmed by comparison of *cytb* and COI sequences with available reference data (Bannikova et al. 2011). On the *cytb* tree, the *Crocidura phuquocensis* haplogroup is the nearest neighbour to the *Crocidura fuliginosa* – *Crocidura dracula* group, although with low bootstrap support (Fig. 2).

Phu Quoc lies very close to mainland Cambodia. The Phu Quoc rainforests belong to the Cardamom Mountain rain forests ecoregion (MacKinnon 1997). Overall, the small mammal fauna of Phu Quoc is similar to that of the Cardamom Mountains (Swan and Kry 2000; Abramov et al. 2007a). Presumably, *Crocidura phuquocensis* (currently known from Phu Quoc only) may also be found in the adjacent mainland.

### Crocidura attenuata

Milne-Edwards, 1872

http://species-id.net/wiki/Crocidura_attenuata

We collected seven specimens of this species from Cat Ba Island. The trapping line (20°48'N, 106°59'E) was located along the west part of tourist trail from the Cat Ba National Park Headquarters to Viet Hai Village. All specimens were caught in pitfall traps set in mixed forest near limestone bare rocks. It is the first record of *Crocidura* from Cat Ba Island.

This is a medium-sized *Crocidura*, with a moderately long tail; tail 79.8% of head and body length, in average. Means and extremes of measurements (in mm) from 5 adults are: head and body length, 74.6 (71–79); tail length, 59.2 (57–62); hind foot length, 13.2 (12–14); weight (n=4), 9.8 (7.3–11.5) g.

Analysis of mitochondrial DNA confirmed that the specimens from Cat Ba belong to *Crocidura attenuata* proper. All the analyzed specimens from Cat Ba (see Fig. 2) formed a single cluster closely related to the group of specimens from northern Vietnam (Ha Giang Province) and southeastern China (Guangxi Province). The genetic distance (*p*-distance) between specimens from Cat Ba/Ha Giang as well as Cat Ba/Guangxi is about 2.1%. The specimen of *Crocidura attenuata* from the more north-eastern region of China (Hunan Province) appears basal among all samples of *Crocidura attenuata* from China and Vietnam. Thus, the genetic distance between two specimens from China (Hunan/Guangxi) is 4.3%, which is nearly the same as the distance between *Crocidura indochinensis*/*Crocidura* sp. AB1. Thus, genetic differentiation of *Crocidura attenuata* is notable and reveals a phylogeographic structure with four haplogroups.

Most authors (Heaney and Timm 1983; Huynh et al. 1994; Hutterer 2005; Kuznetsov 2006; Can et al. 2008; Jenkins et al. 2009) have suggested a wide geographic distribution for *Crocidura attenuata* in Vietnam. However, the recent study of mitochondrial DNA (Bannikova et al. 2011) restricted the distribution of *Crocidura attenuata* proper to the northernmost part of Vietnam. Elsewhere in mainland Vietnam, it is replaced by *Crocidura tanakae*. The latter species was previously considered an endemic of Taiwan (Motokawa et al. 1997, 2001; Hutterer 2005). However, based on mtDNA data analyses, the name *Crocidura tanakae* has been applied to all white-toothed shrews that are genetically similar to the Taiwanese haplogroup. This haplogroup is widely distributed across mainland Asia, including in southern China, Vietnam and Laos (Esselstyn et al. 2009, 2010; Bannikova et al. 2011).

Documentation of *Crocidura attenuata* on Cat Ba Island well corresponds to the proposed species’ distribution confined to the north and east of the Red River (see Bannikova et al. 2011).

## Conclusion

Current distributions and phylogenetic relationships of *Crocidura* species from Vietnamese offshore islands support the hypothesis that shrews may have colonized the islands relatively recently. It is known that the non-volant mammal fauna of these islands was formed during the period when most parts of the modern South China Sea shelf were continental (see also Kuznetsov and Anh 1992; Kuznetsov 2000).

Cat Ba Island is a part of the extended region of the Viet Bac Karst zone, stretching from southeastern China to northeastern Vietnam (Tuyet 1998), and its mammal fauna is similar to that of the adjacent mainland (Abramov and Kruskop 2012). The low level of divergence between mainland populations of *Crocidura attenuata* and that from Cat Ba seem to correspond to recent faunal exchanges in northeastern Vietnam, including Cat Ba Island. *Crocidura phuquocensis*, the only endemic species of white-toothed shrew from coastal islands of Vietnam, is very likely to be found in the Cardamom Mountains of the adjacent mainland, if targeted small mammals surveys are undertaken there.

Sister relationships between the Con Son and Malaysian populations of *Crocidura fuliginosa* suggest that its distribution might have been more extensive in the past. However, data on this species’ occurrence in Southeast Asia are very scarce and doubtful. Morphological characters and genetic variation of the populations from Thailand, Laos, Cambodia and Myanmar referred to as *Crocidura fuliginosa* (Corbet and Hill 1992; Ruedi 1995; Francis 2008) need to be examined in detail.

## Supplementary Material

XML Treatment for Crocidura fuliginosa

XML Treatment for Crocidura phuquocensis

XML Treatment for Crocidura attenuata
